# PxBLAT: an efficient python binding library for BLAT

**DOI:** 10.1186/s12859-024-05844-0

**Published:** 2024-06-19

**Authors:** Yangyang Li, Rendong Yang

**Affiliations:** 1 Department of Urology, Northwestern University Feinberg School of Medicine, 303 E Superior St, Chicago, IL 60611 USA; 2 Robert H. Lurie Comprehensive Cancer Center, Northwestern University Feinberg School of Medicine, 675 N St Clair St, Chicago, IL 60611 USA

**Keywords:** Software libraries, Sequence analysis, BLAT

## Abstract

**Background:**

With the surge in genomic data driven by advancements in sequencing technologies, the demand for efficient bioinformatics tools for sequence analysis has become paramount. BLAST-like alignment tool (BLAT), a sequence alignment tool, faces limitations in performance efficiency and integration with modern programming environments, particularly Python. This study introduces PxBLAT, a Python-based framework designed to enhance the capabilities of BLAT, focusing on usability, computational efficiency, and seamless integration within the Python ecosystem.

**Results:**

PxBLAT demonstrates significant improvements over BLAT in execution speed and data handling, as evidenced by comprehensive benchmarks conducted across various sample groups ranging from 50 to 600 samples. These experiments highlight a notable speedup, reducing execution time compared to BLAT. The framework also introduces user-friendly features such as improved server management, data conversion utilities, and shell completion, enhancing the overall user experience. Additionally, the provision of extensive documentation and comprehensive testing supports community engagement and facilitates the adoption of PxBLAT.

**Conclusions:**

PxBLAT stands out as a robust alternative to BLAT, offering performance and user interaction enhancements. Its development underscores the potential for modern programming languages to improve bioinformatics tools, aligning with the needs of contemporary genomic research. By providing a more efficient, user-friendly tool, PxBLAT has the potential to impact genomic data analysis workflows, supporting faster and more accurate sequence analysis in a Python environment.

**Supplementary Information:**

The online version contains supplementary material available at 10.1186/s12859-024-05844-0.

## Background

The rise of Python as a preferred programming language within bioinformatics is widely acknowledged as a result of its user-friendly nature, extensive libraries, and unparalleled versatility [1]. A variety of libraries have been crafted to augment Python’s interface, thereby amplifying the adaptability and compatibility of bioinformatics tools [2, 3]. For instance, Biopython [3], a preeminent bioinformatics library, furnishes interfaces to tools like Basic Local Alignment Search Tool (BLAST) [4] and Clustal [5]. BLAT, a prominent tool in bioinformatics, is renowned for its speed in genome sequence alignments and serves as a more efficient alternative to BLAST for aligning DNA sequences with the reference genome [6–8]. Furthermore, the unprecedented growth in genome sequencing technologies has significantly increased the availability of genomic data, emphasizing the need for advanced tools in both research and clinical contexts [9, 10]. While BLAT was developed twenty years ago, it continues to be a staple and popular tool in bioinformatics due to its exceptional speed and accuracy. Its enduring relevance is evident across various contexts, from genome sequencing to comparative genomics [11–15].

Despite its popularity and effectiveness, BLAT’s integration is fraught with difficulties, primarily due to its C-based implementation and reliance on Command-Line Interfaces (CLIs), hindering seamless integration into Python projects [16]. Also, executing extensive queries with the BLAT suite leads to inefficiencies when operations are isolated and not executed in batches. Typically, BLAT’s task allocation is sporadic, and intermixed with other tasks. Users generally face a choice: either employ standalone BLAT or integrate *gfServer* with *gfClient* [4]. BLAT’s standard operational model involves initiating *gfServer*, conducting the sequence query through *gfClient*, and subsequently terminating the server after each query. This method becomes highly inefficient for ungrouped, numerous queries as it necessitates the repeated initialization and shutdown of *gfServer*, introducing significant overhead [6]. An optimized approach would entail initiating *gfServer* a single time and leveraging *gfClient* to execute multiple queries. However, the command-line-only access to *gfServer* and *gfClient* complicates this process. This limitation necessitates the management of system calls (like *subprocess* or *os.system*), the handling of intermediate temporary files, and dealing with format conversion, all of which cumulatively degrade performance.

PxBLAT is proposed as a solution that allows for the programmatic use of BLAT, ensuring its smooth integration into new algorithms or analytical pipelines within the Python ecosystem. It acts as a conduit, merging the high-performance capabilities of BLAT with Python’s versatility while ensuring data reproducibility. The primary goal of PxBLAT is to bridge the gap in the current landscape by providing a Python binding library tailored specifically for BLAT, addressing both the efficiency bottlenecks and the ergonomic challenges of its integration.

## Implementation

### Design and architecture

The design of PxBLAT is anchored in the principles of readability and simplicity, fostering an intuitive user interface that minimizes the learning curve for users. In our quest to streamline complexity and amplify both usability and performance, we meticulously extracted the core implementation of BLAT from the broader UCSC Genome Browser (UCSC) codebase, significantly reducing dependency overhead.

We preserved the integrity of the original C codebase while reimplementing key BLAT $\left( \texttt{V}37.1\right)$ utilities such as *faTwoBit*, *gfServer*, and *gfClient* in C++. This strategic choice not only modernizes the code but also enhances maintainability and scalability. The integration of the revamped C++ code with PxBLAT was achieved using Pybind11 [17], a lightweight, seamless method for interfacing C++ and Python.

This approach ensures a direct and efficient interaction with BLAT’s functions, upholding the original performance benchmarks and reliability of BLAT. Simultaneously, it extends the framework’s functionality, aligning it with modern computational standards and making it a robust tool in the bioinformatics toolkit (Table 1).

**Table 1:** Overview of features of PxBLAT compared with BLAT

Feature	PxBLAT	BLAT
Start server	✓	✓
Stop server	✓	✓
Query server	✓	✓
Wait server	✓	✗
Fasta to bit	✓	✗
Bit to fasta	✓	✗
Port retry	✓	✗
Shell completion	✓	✗

This table presents a comprehensive comparison between the features offered by PxBLAT and BLAT. Features are denoted with a ✓ to signify availability and an ✗ to indicate absence. Notably, PxBLAT showcases significant enhancements, particularly in server management (e.g., wait server), data conversion (e.g., fasta to bit, bit to fasta), and enriched user interaction (e.g., shell completion). These advancements firmly establish PxBLAT as a superior and more versatile alternative to the conventional BLAT tool

PxBLAT features CLI utilities crafted through its Application Programming Interfaces (APIs), boasting shell completion for various systems to augment its versatility (Table 1). Recognizing the diverse technological landscape, we provide the library in wheel format compatible with multiple platforms, including Linux x86-64, macOS x86-64, and macOS arm64. This ensures a seamless installation process, free from the complexities of C library dependencies, making it straightforward and user-friendly.

Moreover, PxBLAT utilizes type annotations in its public classes and functions. This not only reinforces code quality and correctness through type checking and static analysis but also enhances the development experience. The annotated types facilitate automatic suggestion and correction of function signatures in development environments, streamlining the coding process.

### APIs of PxBLAT

PxBLAT delivers its query results in alignment with the *QueryResult* class of Biopython [3], enabling seamless manipulation of query outputs (Listing 1). This integration effectively streamlines the post-query workflow, allowing users to leverage the full potential of Biopython in their sequence alignment tasks. Significantly, PxBLAT negates the necessity for intermediate files by conducting all operations in memory. This advancement eliminates the often cumbersome and time-consuming step of data format conversion, enabling users to concentrate on the core aspects of sequence alignment. To enhance user flexibility, the necessity for input and output files has been made optional, aligning with diverse user preferences and workflows.

**Listing 1:**
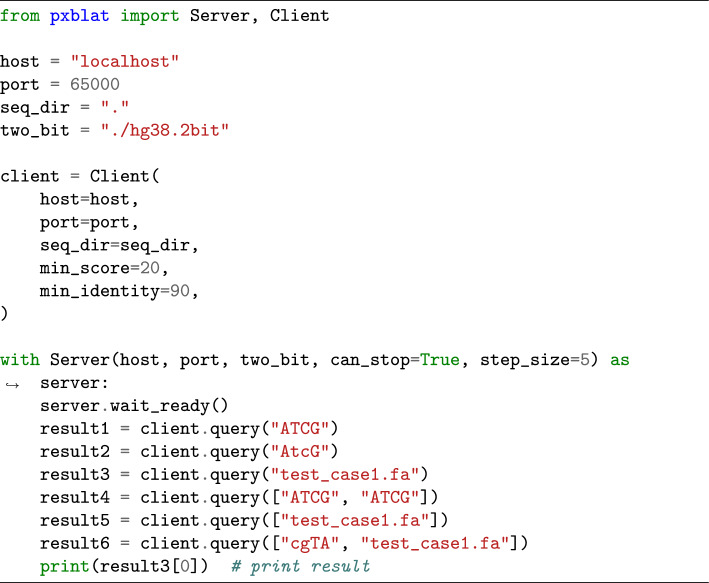
API example. The code snippet shows how to use the API of PxBLAT, and the query result can be iterated. More code examples can be found at https://pxblat.readthedocs.io/en

Recognizing the latency and potential performance bottlenecks induced by system calls, PxBLAT minimizes their usage, thereby streamlining operations and enhancing efficiency. Additionally, PxBLAT simplifies server status retrieval, circumventing the complexities and potential pitfalls of log file manipulation, particularly in concurrent usage scenarios. To further elevate the user experience and operational efficiency, PxBLAT integrates several ergonomic features. These include real-time server readiness checks for alignments, automatic port retries when the default is in use, and the capability to latch onto an already running server if available. These features collectively ensure a smoother, more efficient alignment process, reducing downtime and maximizing productivity.

To facilitate a smooth experience, we offer an extensive range of examples and comprehensive documentation (Listing 1). PxBLAT introduces a robust set of APIs, including the classes *Server* and *Client*, along with a suite of functions designed to replicate the capabilities of the BLAT suite. These classes mirror the utilities of the CLI tools *gfServer* and *gfClient*, respectively, but with added flexibility to accommodate a wider range of user requirements. Key functions such as *start_server*, *query_server*, *status_server*, *fa2twobit*, and *twobit2fa* are provided to cater to diverse usage scenarios. Rigorous testing and development protocols, incorporating Continuous Integration (CI) and Continuous Development (CD), have been employed to ensure high code quality and reliability.

## Results

### Performance on real datasets

**Fig. 1:**
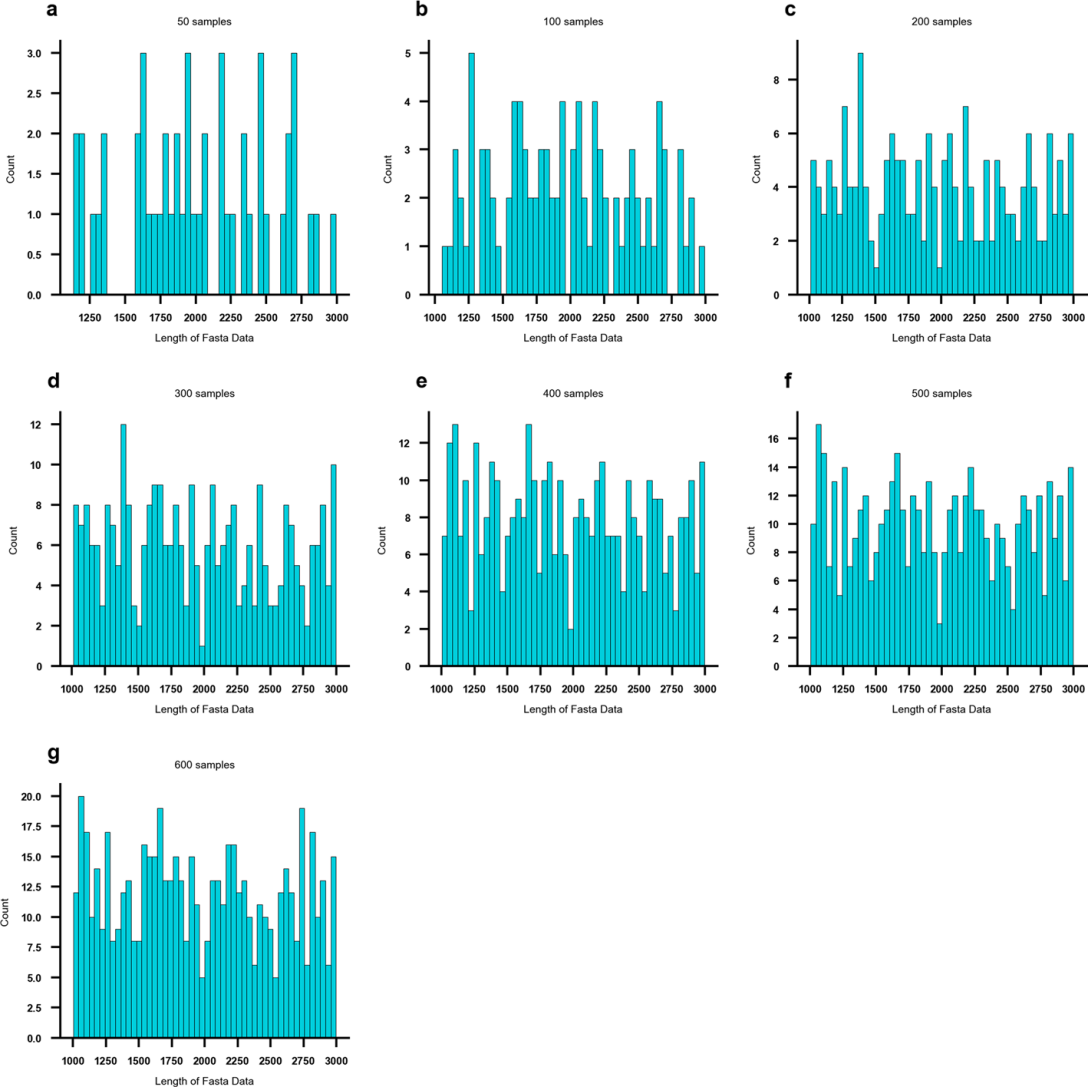
Sequence length distribution in real datasets. This figure illustrates the distribution of fasta sequence lengths across different sample sets. The $x$ axis represents the sequence length, while the $y$ axis denotes the count of each length. **a** Distribution of a set of 50.00 samples. **b** Distribution of a set of 100.00 samples. **c** Distribution of a set of 200.00 samples. **d** Distribution of a set of 300.00 samples. **e** Distribution of a set of 400.00 samples. **f** Distribution of a set of 500.00 samples. **g** Distribution of a set of 600.00 samples

The performance of PxBLAT was rigorously benchmarked against BLAT $\left( \texttt{V}37.1\right)$, utilizing eight distinct sample sets of FASTA files. Each set comprised a group of samples, ranging from 50 to 600 samples per set. The datasets are sampled from chromosome 20 of the genome of *Homo sapiens* (hg38), with each sample containing a single sequence. These sequences varied in length from 1000.00 bp to 3000.00 bp, encompassing a spectrum of typical use-case scenarios (Fig 1).

To ascertain the accuracy and reliability of PxBLAT, we conducted a comparative analysis of the High-Scoring Pairs (HSPs) generated by both BLAT and PxBLAT for each sample. This side-by-side comparison indicated a complete alignment between the HSPs generated by PxBLAT and BLAT, validating the precision of PxBLAT’s results (S2 Table).

**Fig. 2:**
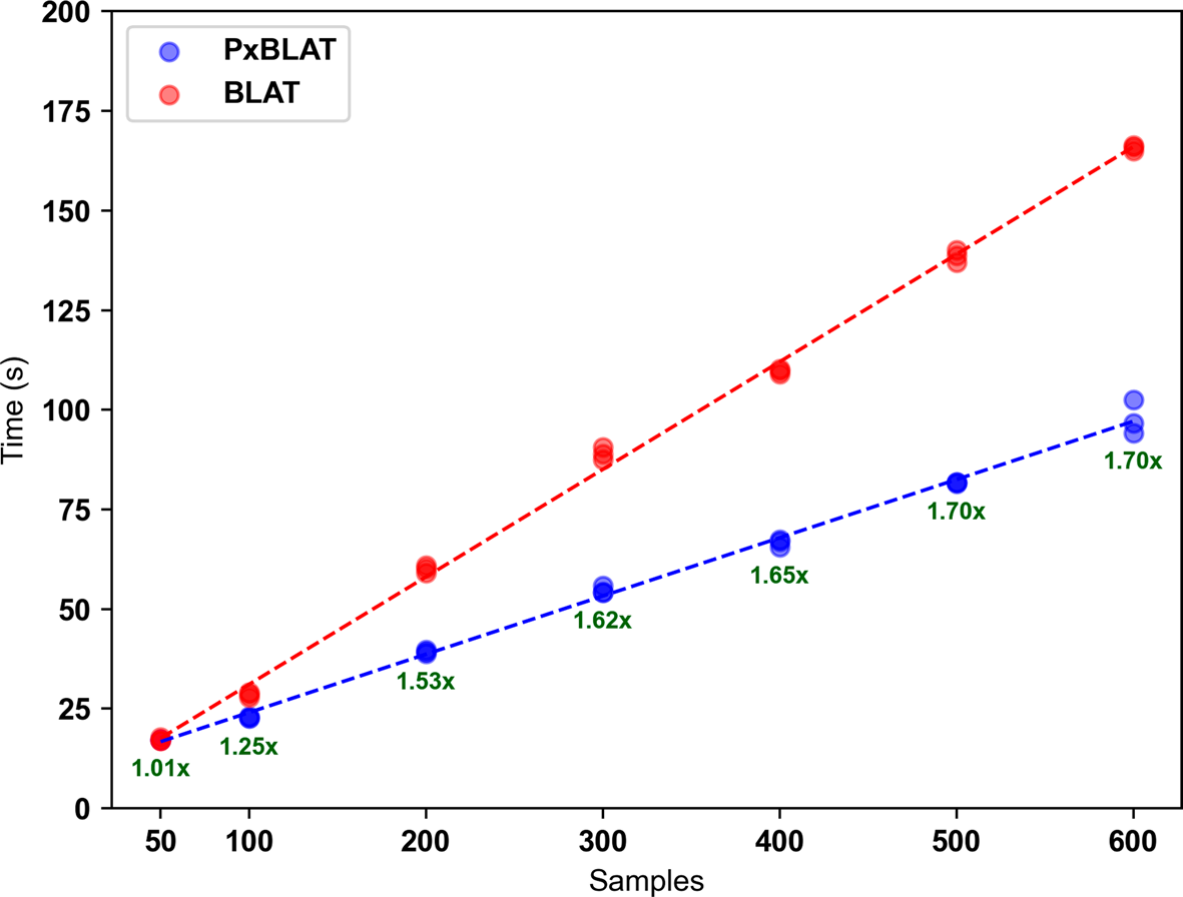
Performance comparison between BLAT and PxBLAT. This figure quantifies the performance of BLAT (indicated by red points) and PxBLAT (indicated by blue points) across various data sets, with the *x* axis categorizing the number of samples in the sets and the *y* axis detailing the execution time in seconds. Each group encapsulates the results of three independent experiments. Trend lines, depicted in red for BLAT and blue for PxBLAT, illustrate the general performance pattern for each tool. Notably, the green text highlights the speedup achieved by PxBLAT, calculated as the ratio of the execution time ($\text {time}_{_{\text {blat}}} / \text {time}_{_{\text {pxblat}}}$), underscoring the efficiency gains of PxBLAT relative to BLAT

The benchmarking process was carried out on an Apple M1 Pro running macOS 13.4.1 (arm64). For launching BLAT, system calls were utilized, and the execution time was measured using the time library. Each set of FASTA files underwent three experimental runs, facilitating a comprehensive assessment of performance. The results highlighted the efficiency of PxBLAT, with observed speedups ranging from 1.00 to 1.77 times compared to the BLAT execution (Fig 2).

In summary, PxBLAT demonstrates significant advantages in terms of execution time reduction. These findings underscore its utility as a substantial improvement over the BLAT, reinforcing its value within the bioinformatics toolkit.

## Conclusion

In conclusion, PxBLAT is a robust, efficient, and user-friendly Python binding library designed to enhance the capabilities of BLAT. It is freely available for non-commerial users. Its seamless integration with the Python ecosystem, coupled with its enhanced performance, underscores its potential to impact genomic data analysis workflows. Overall, PxBLAT provides a comprehensive set of features, including server management, data conversion utilities, and shell completion, to enhance the user experience.

We plan to explore the implementation of a distributed service architecture and multi-tenant management support, despite the challenges posed by the BLAT architecture. Additionally, incorporating a dynamic BLAT server is another future direction to further enhance the performance of PxBLAT. These future enhancements aim to improve the performance and scalability of PxBLAT, ensuring it remains a valuable tool for the bioinformatics community.

## Availability and requirements

Project name: PxBLAT

Project home page: https://github.com/ylab-hi/pxblat

Operating system(s): Linux, Mac OS X

Programming language: C, C++, Python (version 3.9.0 or higher).

License: The source code and executables are freely available for academic, nonprofit, and personal use. Commercial licensing information is available on the Kent Informatics website (http://www.kentinformatics.com).

Any restrictions to use by non-academics: license needed

## Supplementary Information

Supplementary Material 1.

## Data Availability

The PxBLAT, along with the source code, is publicly available in the GitHub repository at https://github.com/ylab-hi/pxblat. The documentation is available at ReadtheDocs https://pxblat.readthedocs.io/en/latest/. The script for benchmarking is available at *tests/test_result.py* in the repository. The testing dataset is available at the GitHub repository https://github.com/ylab-hi/pxblat. The path of the testing dataset is *benchmark/fas*.

## Abbreviations

API: Application Programming Interface
BLAST: Basic Local Alignment Search Tool
BLAT: BLAST-like alignment tool
CD: Continuous Development
CI: Continuous Integration
CLI: Command-Line Interface
HSP: High-Scoring Pair
UCSC: UCSC Genome Browser
