# Supplementary material for: PxBLAT: an efficient python binding library for BLAT
Source: BMC Bioinformatics. 2024 Jun 19;25:219. doi: 10.1186/s12859-024-05844-0 (PMC11549839; doi:10.1186/s12859-024-05844-0)
Supplement: Supplementary file 1 — Supplementary Material 1. [file 12859_2024_5844_MOESM1_ESM.pdf]

Supplementary information.

S1 Table.

Performance comparison between PxBLAT and BLAT. This table illustrates the performance of both PxBLAT and BLAT across data sets containing 50.00, 100.00, 200.00, 300.00, 400.00, 500.00, and 600.00 samples. For each data set, three independent experiments are conducted to ensure robust performance evaluation. The efficiency of PxBLAT relative to BLAT is quantified through the speedup metric, calculated as the ratio of the execution time ($\text{time}_{\text{blat}}/\text{time}_{\text{pxblat}}$). This comparison highlights the computational advantages of PxBLAT in terms of processing speed.

Samples	PxBLAT (s)	BLAT (s)	Speedup
50.00	17.10	17.03	1.00
50.00	17.09	17.01	1.00
50.00	17.10	17.72	1.04
100.00	22.75	28.61	1.26
100.00	23.12	29.10	1.26
100.00	22.53	27.90	1.24
200.00	39.31	59.03	1.50
200.00	38.86	60.20	1.55
200.00	39.80	60.87	1.53
300.00	55.89	90.68	1.62
300.00	54.34	88.88	1.64
300.00	54.15	87.46	1.62
400.00	65.50	110.20	1.68
400.00	67.02	109.06	1.63
400.00	67.46	109.79	1.63
500.00	81.56	140.13	1.72
500.00	81.77	138.66	1.70
500.00	81.73	137.02	1.68
600.00	96.62	165.87	1.72
600.00	102.55	164.93	1.61
600.00	94.21	166.38	1.77

S2 Table.

HSP comparison between PxBLAT and BLAT. This table, including 600.00 samples in total, presents a comparison of the HSPs generated by BLAT and PxBLAT for each sample. The column Sample lists the name of the sample, and the columns BLAT and PxBLAT, respectively denote the number of HSPs generated by BLAT and PxBLAT.

Sample	BLAT	PxBLAT
chr20-11648866-11650925	130.00	130.00
chr20-29850079-29852595	1.00	1.00
chr20-7493878-7496145	26.00	26.00
chr20-30152074-30153892	3.00	3.00
chr20-1140095-1141788	22.00	22.00
chr20-17520690-17523557	14.00	14.00
chr20-41374266-41376291	12.00	12.00
chr20-63240773-63243242	64.00	64.00
chr20-59286740-59288091	13.00	13.00
chr20-8061734-8063508	10.00	10.00
chr20-34429296-34431248	41.00	41.00
chr20-27977914-27979241	22.00	22.00
chr20-11695455-11697457	20.00	20.00
chr20-41792348-41794797	131.00	131.00
chr20-25858004-25860351	37.00	37.00
chr20-31404034-31407027	25.00	25.00
chr20-15852904-15855300	65.00	65.00
chr20-29917054-29919227	18.00	18.00
chr20-45175162-45177033	2.00	2.00
chr20-43937342-43939507	58.00	58.00
chr20-17073859-17076499	48.00	48.00
chr20-19823346-19824950	32.00	32.00
chr20-28295597-28298301	23.00	23.00
chr20-15826504-15829187	26.00	26.00
chr20-54683378-54684991	47.00	47.00
chr20-55475241-55476377	18.00	18.00
chr20-57949934-57952283	74.00	74.00
chr20-14442978-14444604	35.00	35.00
chr20-51293808-51295630	38.00	38.00
chr20-49194155-49196823	165.00	165.00
chr20-34696351-34697788	43.00	43.00
chr20-16044321-16045712	48.00	48.00
chr20-45309757-45312553	39.00	39.00
chr20-21063232-21065145	26.00	26.00
chr20-434138-436331	174.00	174.00
chr20-62110486-62112604	167.00	167.00
chr20-15866914-15868171	15.00	15.00
chr20-62343940-62345043	69.00	69.00
chr20-37168356-37170160	34.00	34.00
chr20-15699683-15701260	15.00	15.00
chr20-44826183-44827333	152.00	152.00
chr20-41538435-41540074	43.00	43.00

Continued on next page

Sample	BLAT	PxBLAT
chr20-56032843-56034378	18.00	18.00
chr20-36813618-36816516	21.00	21.00
chr20-18637848-18640751	68.00	68.00
chr20-56294086-56296598	27.00	27.00
chr20-19514094-19515343	2.00	2.00
chr20-33893321-33895564	126.00	126.00
chr20-22282380-22283429	4.00	4.00
chr20-39910629-39912740	84.00	84.00
chr20-40898874-40901542	27.00	27.00
chr20-7536153-7537696	15.00	15.00
chr20-4818626-4819858	60.00	60.00
chr20-64316707-64319236	24.00	24.00
chr20-2960440-2961833	119.00	119.00
chr20-2519249-2521676	46.00	46.00
chr20-11596878-11599706	68.00	68.00
chr20-30829536-30831739	16.00	16.00
chr20-45188854-45190558	3.00	3.00
chr20-47238745-47241145	14.00	14.00
chr20-10538965-10540232	83.00	83.00
chr20-11996320-11998343	87.00	87.00
chr20-23440919-23442976	47.00	47.00
chr20-22451495-22453568	4.00	4.00
chr20-39709672-39711911	23.00	23.00
chr20-29782708-29784088	8.00	8.00
chr20-7911434-7913178	9.00	9.00
chr20-1508550-1511129	193.00	193.00
chr20-41451172-41452837	6.00	6.00
chr20-4003988-4006545	29.00	29.00
chr20-10921197-10923149	5.00	5.00
chr20-36789016-36790500	186.00	186.00
chr20-9309222-9310479	157.00	157.00
chr20-52532795-52534564	9.00	9.00
chr20-42081563-42082983	4.00	4.00
chr20-12605319-12606971	2.00	2.00
chr20-29389010-29390598	149.00	149.00
chr20-8128132-8130266	170.00	170.00
chr20-11399422-11401237	12.00	12.00
chr20-33251227-33252414	2.00	2.00
chr20-3342256-3343645	92.00	92.00
chr20-38869283-38872088	60.00	60.00
chr20-7649152-7650444	60.00	60.00
chr20-13021673-13023769	58.00	58.00

Continued on next page

Sample	BLAT	PxBLAT
chr20-3971117-3972667	32.00	32.00
chr20-26232703-26234929	61.00	61.00
chr20-23063517-23064745	7.00	7.00
chr20-39079691-39080763	26.00	26.00
chr20-23882126-23884119	92.00	92.00
chr20-3170215-3172728	44.00	44.00
chr20-49421601-49423499	35.00	35.00
chr20-55380779-55383730	204.00	204.00
chr20-45794728-45796900	164.00	164.00
chr20-15159613-15161221	9.00	9.00
chr20-62378279-62380174	184.00	184.00
chr20-6934771-6936584	116.00	116.00
chr20-59538484-59541329	8.00	8.00
chr20-43661982-43664714	26.00	26.00
chr20-36707842-36709247	85.00	85.00
chr20-33898085-33900435	65.00	65.00
chr20-18062688-18064265	46.00	46.00
chr20-29490152-29491491	23.00	23.00
chr20-30806959-30809757	0.00	0.00
chr20-11837593-11840377	40.00	40.00
chr20-28934655-28937276	1.00	1.00
chr20-7689471-7691553	18.00	18.00
chr20-1746811-1749803	9.00	9.00
chr20-1261408-1262588	16.00	16.00
chr20-5757029-5759548	29.00	29.00
chr20-5361957-5364278	107.00	107.00
chr20-37305589-37308001	21.00	21.00
chr20-53702835-53705300	148.00	148.00
chr20-7447290-7449191	16.00	16.00
chr20-36626015-36629010	150.00	150.00
chr20-48184357-48185678	2.00	2.00
chr20-4634043-4636057	9.00	9.00
chr20-21292878-21295830	179.00	179.00
chr20-52551672-52553086	7.00	7.00
chr20-55410142-55411547	55.00	55.00
chr20-15164296-15166007	21.00	21.00
chr20-60294852-60297789	197.00	197.00
chr20-47112993-47114241	38.00	38.00
chr20-33645643-33648541	62.00	62.00
chr20-57099663-57101555	91.00	91.00
chr20-32443012-32444528	1.00	1.00
chr20-54597242-54598325	31.00	31.00

Continued on next page

Sample	BLAT	PxBLAT
chr20-28168758-28171348	20.00	20.00
chr20-14255274-14256294	211.00	211.00
chr20-22858139-22859797	8.00	8.00
chr20-42977157-42978222	11.00	11.00
chr20-3890553-3891667	46.00	46.00
chr20-10043505-10044872	33.00	33.00
chr20-47292153-47293840	50.00	50.00
chr20-30056354-30058719	0.00	0.00
chr20-23406092-23408775	15.00	15.00
chr20-44245002-44246392	36.00	36.00
chr20-30999482-31002350	1.00	1.00
chr20-11990150-11991239	3.00	3.00
chr20-3810992-3812466	197.00	197.00
chr20-26419101-26422061	0.00	0.00
chr20-22091287-22094021	119.00	119.00
chr20-47477509-47479589	59.00	59.00
chr20-13750647-13753070	44.00	44.00
chr20-61483678-61484972	2.00	2.00
chr20-57584630-57586794	13.00	13.00
chr20-17904728-17906925	34.00	34.00
chr20-28086098-28087474	19.00	19.00
chr20-21317295-21318548	51.00	51.00
chr20-42758324-42759488	193.00	193.00
chr20-23580801-23581810	23.00	23.00
chr20-58929386-58930631	22.00	22.00
chr20-6605062-6607136	79.00	79.00
chr20-32987534-32989963	169.00	169.00
chr20-22493043-22494805	22.00	22.00
chr20-54190709-54192035	24.00	24.00
chr20-48343887-48346044	2.00	2.00
chr20-10262876-10265171	189.00	189.00
chr20-35374558-35376267	67.00	67.00
chr20-39018648-39021540	83.00	83.00
chr20-37785924-37788264	202.00	202.00
chr20-64334961-64337760	0.00	0.00
chr20-12575681-12578297	14.00	14.00
chr20-23374273-23375283	1.00	1.00
chr20-14278920-14280350	40.00	40.00
chr20-42778102-42780755	9.00	9.00
chr20-52810557-52811573	83.00	83.00
chr20-52564945-52566606	22.00	22.00
chr20-10598821-10601808	32.00	32.00

Continued on next page

Sample	BLAT	PxBLAT
chr20-1172826-1175431	87.00	87.00
chr20-3347653-3350627	44.00	44.00
chr20-64176870-64179763	43.00	43.00
chr20-42829128-42830258	100.00	100.00
chr20-40623645-40625957	187.00	187.00
chr20-27009229-27011878	24.00	24.00
chr20-14732847-14734481	95.00	95.00
chr20-12698635-12699679	2.00	2.00
chr20-13328590-13330614	13.00	13.00
chr20-22912395-22915205	12.00	12.00
chr20-35339375-35342113	48.00	48.00
chr20-19431509-19433187	28.00	28.00
chr20-28727730-28729750	16.00	16.00
chr20-39515806-39517118	2.00	2.00
chr20-21857448-21859310	209.00	209.00
chr20-44751640-44753567	19.00	19.00
chr20-47371415-47374127	45.00	45.00
chr20-24140371-24142959	6.00	6.00
chr20-54083025-54084528	41.00	41.00
chr20-51775800-51777458	46.00	46.00
chr20-59400225-59402632	30.00	30.00
chr20-57144627-57147621	47.00	47.00
chr20-17690928-17692128	6.00	6.00
chr20-31238605-31240139	4.00	4.00
chr20-29259215-29262074	4.00	4.00
chr20-39132690-39134466	34.00	34.00
chr20-44674003-44676622	31.00	31.00
chr20-34435089-34436815	13.00	13.00
chr20-11085268-11087065	27.00	27.00
chr20-42010931-42012642	10.00	10.00
chr20-7483012-7484115	1.00	1.00
chr20-39873233-39875635	207.00	207.00
chr20-32029134-32032031	93.00	93.00
chr20-38052616-38054138	32.00	32.00
chr20-2602976-2605324	19.00	19.00
chr20-42169915-42172757	15.00	15.00
chr20-62125729-62128471	19.00	19.00
chr20-2162753-2164165	5.00	5.00
chr20-34630972-34632436	113.00	113.00
chr20-6611620-6614440	152.00	152.00
chr20-19300360-19302642	6.00	6.00
chr20-25189802-25191365	154.00	154.00

Continued on next page

Sample	BLAT	PxBLAT
chr20-15951964-15953450	26.00	26.00
chr20-47285001-47287102	52.00	52.00
chr20-22548369-22549974	9.00	9.00
chr20-24725480-24727693	52.00	52.00
chr20-55633941-55636718	5.00	5.00
chr20-30324027-30326285	30.00	30.00
chr20-34789703-34791591	27.00	27.00
chr20-22886111-22887126	5.00	5.00
chr20-52373722-52374925	52.00	52.00
chr20-62594615-62596270	61.00	61.00
chr20-23567798-23569994	5.00	5.00
chr20-52746782-52748970	26.00	26.00
chr20-63919107-63920159	20.00	20.00
chr20-37603411-37606081	21.00	21.00
chr20-45549091-45551404	167.00	167.00
chr20-24122821-24124880	2.00	2.00
chr20-39384970-39386746	47.00	47.00
chr20-56953750-56954962	11.00	11.00
chr20-27265832-27268665	27.00	27.00
chr20-989008-991475	96.00	96.00
chr20-13233506-13234845	9.00	9.00
chr20-30767954-30769153	0.00	0.00
chr20-54079315-54081921	56.00	56.00
chr20-22276591-22279281	202.00	202.00
chr20-32344273-32347087	26.00	26.00
chr20-14761627-14763228	15.00	15.00
chr20-24471803-24474607	7.00	7.00
chr20-22305788-22308754	43.00	43.00
chr20-63927315-63929626	54.00	54.00
chr20-33534721-33537439	158.00	158.00
chr20-53020990-53022920	33.00	33.00
chr20-44701412-44703325	34.00	34.00
chr20-63523339-63524524	190.00	190.00
chr20-39750571-39752199	50.00	50.00
chr20-47879542-47880820	162.00	162.00
chr20-12949055-12950079	1.00	1.00
chr20-37737796-37740734	26.00	26.00
chr20-17600492-17603407	68.00	68.00
chr20-40848143-40849212	2.00	2.00
chr20-33135173-33136880	131.00	131.00
chr20-39118192-39119260	4.00	4.00
chr20-27690465-27692366	23.00	23.00

Continued on next page

Sample	BLAT	PxBLAT
chr20-37858061-37860321	49.00	49.00
chr20-12405605-12407882	6.00	6.00
chr20-57386121-57387852	70.00	70.00
chr20-34902889-34905611	39.00	39.00
chr20-52070637-52073331	35.00	35.00
chr20-40779660-40781751	47.00	47.00
chr20-31098450-31100717	3.00	3.00
chr20-29969458-29971235	18.00	18.00
chr20-13789470-13791842	152.00	152.00
chr20-26814485-26817257	22.00	22.00
chr20-40740691-40742983	29.00	29.00
chr20-49955693-49958425	183.00	183.00
chr20-37813305-37814584	9.00	9.00
chr20-18255943-18258048	64.00	64.00
chr20-63109432-63111043	34.00	34.00
chr20-14211039-14213272	9.00	9.00
chr20-17100441-17103140	9.00	9.00
chr20-2866996-2869328	38.00	38.00
chr20-53703835-53705398	158.00	158.00
chr20-51431118-51433743	56.00	56.00
chr20-6192786-6193790	196.00	196.00
chr20-10748356-10751019	35.00	35.00
chr20-33974548-33976407	44.00	44.00
chr20-32270385-32273111	20.00	20.00
chr20-36902282-36904506	28.00	28.00
chr20-198832-201190	40.00	40.00
chr20-7927314-7928574	27.00	27.00
chr20-17899461-17900502	58.00	58.00
chr20-11120841-11122366	23.00	23.00
chr20-59407661-59409193	27.00	27.00
chr20-35649824-35652625	5.00	5.00
chr20-30688628-30690580	48.00	48.00
chr20-49466264-49467309	5.00	5.00
chr20-63692672-63694078	2.00	2.00
chr20-12570747-12573421	5.00	5.00
chr20-8512304-8514491	20.00	20.00
chr20-24204303-24205314	2.00	2.00
chr20-11639430-11641175	20.00	20.00
chr20-26938330-26940788	24.00	24.00
chr20-17587799-17589501	209.00	209.00
chr20-26384682-26386149	23.00	23.00
chr20-46308421-46310414	41.00	41.00

Continued on next page

Sample	BLAT	PxBLAT
chr20-30072951-30074554	0.00	0.00
chr20-44079162-44081096	23.00	23.00
chr20-21475551-21476686	50.00	50.00
chr20-13972488-13974627	44.00	44.00
chr20-45697903-45699479	88.00	88.00
chr20-24838480-24839925	11.00	11.00
chr20-7742386-7745111	4.00	4.00
chr20-56522851-56524815	12.00	12.00
chr20-22497057-22499773	16.00	16.00
chr20-51998729-52000293	22.00	22.00
chr20-38767581-38768689	5.00	5.00
chr20-19863311-19865822	56.00	56.00
chr20-9295230-9297764	226.00	226.00
chr20-15012779-15015584	8.00	8.00
chr20-5041846-5044587	56.00	56.00
chr20-580210-582022	35.00	35.00
chr20-15328579-15331324	42.00	42.00
chr20-49481967-49483214	17.00	17.00
chr20-5260817-5262044	5.00	5.00
chr20-23340929-23343084	102.00	102.00
chr20-2269716-2270774	189.00	189.00
chr20-46399947-46401667	31.00	31.00
chr20-10815100-10817410	32.00	32.00
chr20-45864818-45866440	53.00	53.00
chr20-45489949-45492069	35.00	35.00
chr20-21302718-21304312	1.00	1.00
chr20-9931834-9933992	5.00	5.00
chr20-56295194-56296721	5.00	5.00
chr20-38225781-38227971	18.00	18.00
chr20-18585659-18587381	35.00	35.00
chr20-16940912-16942721	39.00	39.00
chr20-3758200-3759977	4.00	4.00
chr20-11807056-11808124	3.00	3.00
chr20-127575-129488	93.00	93.00
chr20-40755444-40756701	5.00	5.00
chr20-21011682-21013735	2.00	2.00
chr20-10119043-10120171	59.00	59.00
chr20-1313128-1315640	18.00	18.00
chr20-11712977-11715178	142.00	142.00
chr20-34597997-34600726	28.00	28.00
chr20-53048517-53050101	29.00	29.00
chr20-15161566-15163488	8.00	8.00

Continued on next page

Sample	BLAT	PxBLAT
chr20-15137364-15139959	203.00	203.00
chr20-60342420-60345056	94.00	94.00
chr20-55169861-55170982	156.00	156.00
chr20-24314101-24316265	27.00	27.00
chr20-53183314-53186148	16.00	16.00
chr20-43887385-43889661	75.00	75.00
chr20-59470391-59471545	1.00	1.00
chr20-53159093-53161302	35.00	35.00
chr20-5895129-5896312	26.00	26.00
chr20-25292499-25293803	3.00	3.00
chr20-34161145-34162369	41.00	41.00
chr20-45868564-45870103	74.00	74.00
chr20-4887453-4889888	3.00	3.00
chr20-39559840-39561722	9.00	9.00
chr20-33221436-33223116	56.00	56.00
chr20-755464-758088	35.00	35.00
chr20-1111176-1112831	46.00	46.00
chr20-2802607-2803838	36.00	36.00
chr20-38997152-38999498	22.00	22.00
chr20-5049946-5052675	35.00	35.00
chr20-48767423-48769196	48.00	48.00
chr20-2659893-2661452	56.00	56.00
chr20-49121260-49124086	21.00	21.00
chr20-54247731-54250009	75.00	75.00
chr20-8334685-8337480	34.00	34.00
chr20-26427340-26428991	0.00	0.00
chr20-1762478-1764560	46.00	46.00
chr20-53286968-53289954	23.00	23.00
chr20-23613637-23615158	53.00	53.00
chr20-7135001-7136753	88.00	88.00
chr20-59872605-59875175	5.00	5.00
chr20-33944307-33946354	27.00	27.00
chr20-29344297-29347205	8.00	8.00
chr20-28334593-28336360	22.00	22.00
chr20-49230825-49232042	66.00	66.00
chr20-48044237-48045631	3.00	3.00
chr20-26227485-26229228	5.00	5.00
chr20-26487906-26489572	25.00	25.00
chr20-11596974-11599728	65.00	65.00
chr20-45661385-45663135	194.00	194.00
chr20-23562380-23564047	2.00	2.00
chr20-5735736-5738602	12.00	12.00

Continued on next page

Sample	BLAT	PxBLAT
chr20-31727237-31729024	39.00	39.00
chr20-36152090-36154966	151.00	151.00
chr20-1018906-1020553	11.00	11.00
chr20-9821106-9823172	75.00	75.00
chr20-52034533-52037144	85.00	85.00
chr20-15008181-15010280	66.00	66.00
chr20-41851737-41853531	6.00	6.00
chr20-29408974-29411107	20.00	20.00
chr20-10955881-10957008	1.00	1.00
chr20-8970270-8971572	18.00	18.00
chr20-2350436-2352589	200.00	200.00
chr20-35494115-35496325	37.00	37.00
chr20-16994461-16996880	203.00	203.00
chr20-1121638-1123536	20.00	20.00
chr20-10539946-10542788	76.00	76.00
chr20-31981394-31984035	24.00	24.00
chr20-31059406-31062015	9.00	9.00
chr20-23005241-23006490	85.00	85.00
chr20-27678668-27680740	22.00	22.00
chr20-23089029-23090440	1.00	1.00
chr20-18766639-18768846	74.00	74.00
chr20-35270491-35271927	24.00	24.00
chr20-32671767-32673927	46.00	46.00
chr20-5469364-5470405	1.00	1.00
chr20-44586665-44588074	3.00	3.00
chr20-19751371-19752469	50.00	50.00
chr20-11475039-11477338	4.00	4.00
chr20-52930875-52933353	52.00	52.00
chr20-23941121-23942933	189.00	189.00
chr20-10612667-10615654	16.00	16.00
chr20-39220431-39222072	6.00	6.00
chr20-24056618-24059198	190.00	190.00
chr20-63753892-63756615	196.00	196.00
chr20-64298054-64301011	16.00	16.00
chr20-43632072-43634462	46.00	46.00
chr20-29402266-29403661	49.00	49.00
chr20-34611441-34612505	39.00	39.00
chr20-16219627-16222599	19.00	19.00
chr20-47623287-47624373	18.00	18.00
chr20-37572653-37574987	20.00	20.00
chr20-57328038-57329145	159.00	159.00
chr20-54255861-54257299	11.00	11.00

Continued on next page

Sample	BLAT	PxBLAT
chr20-17523656-17525455	3.00	3.00
chr20-3054439-3055812	26.00	26.00
chr20-51095602-51096740	8.00	8.00
chr20-26295259-26296843	6.00	6.00
chr20-16956946-16959866	4.00	4.00
chr20-59871467-59873730	18.00	18.00
chr20-12972124-12973571	9.00	9.00
chr20-64187505-64190371	7.00	7.00
chr20-20036801-20038441	54.00	54.00
chr20-52361579-52363650	49.00	49.00
chr20-48763637-48765211	21.00	21.00
chr20-29272745-29274078	3.00	3.00
chr20-6596603-6597647	1.00	1.00
chr20-25092945-25094053	38.00	38.00
chr20-23976043-23978934	34.00	34.00
chr20-33528290-33530524	183.00	183.00
chr20-45968564-45971166	41.00	41.00
chr20-29195839-29197450	17.00	17.00
chr20-12991634-12993772	18.00	18.00
chr20-7287285-7289523	45.00	45.00
chr20-12780972-12782351	20.00	20.00
chr20-17273569-17275885	46.00	46.00
chr20-53404329-53405379	8.00	8.00
chr20-32199416-32201827	159.00	159.00
chr20-56899277-56900938	64.00	64.00
chr20-21026086-21027263	198.00	198.00
chr20-29495766-29497356	5.00	5.00
chr20-46509725-46510768	176.00	176.00
chr20-44370323-44372070	36.00	36.00
chr20-34807421-34810326	128.00	128.00
chr20-29310629-29312542	80.00	80.00
chr20-1861413-1862970	10.00	10.00
chr20-13425759-13427299	2.00	2.00
chr20-40797946-40799834	14.00	14.00
chr20-9134988-9136715	38.00	38.00
chr20-6129194-6130506	20.00	20.00
chr20-32328850-32331341	40.00	40.00
chr20-37816931-37818838	30.00	30.00
chr20-43643216-43645801	82.00	82.00
chr20-57951819-57954389	39.00	39.00
chr20-38495333-38497995	69.00	69.00
chr20-23579562-23580734	13.00	13.00

Continued on next page

Sample	BLAT	PxBLAT
chr20-32476687-32477738	89.00	89.00
chr20-46657968-46660258	17.00	17.00
chr20-44029371-44030373	1.00	1.00
chr20-19040746-19042992	24.00	24.00
chr20-41547378-41548428	5.00	5.00
chr20-31338848-31340507	33.00	33.00
chr20-50733531-50736468	30.00	30.00
chr20-23264110-23266210	46.00	46.00
chr20-59975068-59976189	25.00	25.00
chr20-40116512-40117628	2.00	2.00
chr20-34348380-34351135	191.00	191.00
chr20-53857114-53859581	90.00	90.00
chr20-24510328-24512009	40.00	40.00
chr20-2400648-2401902	51.00	51.00
chr20-41951417-41953958	8.00	8.00
chr20-10519505-10521311	173.00	173.00
chr20-38229341-38232239	39.00	39.00
chr20-1961461-1962512	4.00	4.00
chr20-4493702-4496293	164.00	164.00
chr20-39058190-39059640	65.00	65.00
chr20-11840010-11842763	159.00	159.00
chr20-51385931-51387697	2.00	2.00
chr20-32743241-32746228	61.00	61.00
chr20-36043748-36044868	4.00	4.00
chr20-47940674-47941937	27.00	27.00
chr20-22906294-22908788	8.00	8.00
chr20-46563742-46565560	42.00	42.00
chr20-34970963-34972526	22.00	22.00
chr20-46825075-46827145	11.00	11.00
chr20-6428315-6430827	188.00	188.00
chr20-42217777-42219934	16.00	16.00
chr20-19913134-19914585	34.00	34.00
chr20-37718004-37720318	152.00	152.00
chr20-51873445-51875614	85.00	85.00
chr20-21185966-21187683	41.00	41.00
chr20-36715891-36717659	71.00	71.00
chr20-25706788-25709614	123.00	123.00
chr20-50918346-50919658	55.00	55.00
chr20-19216584-19217715	110.00	110.00
chr20-60913180-60914343	27.00	27.00
chr20-40445916-40447302	2.00	2.00
chr20-39471792-39473038	200.00	200.00

Continued on next page

Sample	BLAT	PxBLAT
chr20-36763453-36764650	82.00	82.00
chr20-58906803-58909272	170.00	170.00
chr20-24358042-24359864	9.00	9.00
chr20-5054910-5055935	113.00	113.00
chr20-39718409-39719767	60.00	60.00
chr20-48995874-48997427	47.00	47.00
chr20-11060084-11062288	61.00	61.00
chr20-3041121-3043902	46.00	46.00
chr20-11729530-11731335	4.00	4.00
chr20-6876021-6878545	44.00	44.00
chr20-60459034-60461357	22.00	22.00
chr20-50558110-50560711	68.00	68.00
chr20-1551191-1553332	2.00	2.00
chr20-23189149-23191563	50.00	50.00
chr20-46682255-46683957	5.00	5.00
chr20-46693319-46694364	56.00	56.00
chr20-50170226-50172189	11.00	11.00
chr20-63698924-63701762	5.00	5.00
chr20-62756096-62757580	69.00	69.00
chr20-55371351-55373202	30.00	30.00
chr20-61650045-61652414	3.00	3.00
chr20-33091750-33093858	24.00	24.00
chr20-61216684-61218692	7.00	7.00
chr20-47978091-47980293	85.00	85.00
chr20-16870384-16872626	14.00	14.00
chr20-55265469-55267149	6.00	6.00
chr20-42713744-42715938	38.00	38.00
chr20-20608914-20611491	36.00	36.00
chr20-59966328-59967826	57.00	57.00
chr20-29829808-29831310	8.00	8.00
chr20-55321262-55323731	34.00	34.00
chr20-38905281-38906801	57.00	57.00
chr20-5081835-5084428	35.00	35.00
chr20-12399681-12402397	19.00	19.00
chr20-15787670-15789236	10.00	10.00
chr20-61849267-61851471	112.00	112.00
chr20-42224821-42226456	8.00	8.00
chr20-2200973-2202184	2.00	2.00
chr20-44016368-44018208	138.00	138.00
chr20-42732744-42735046	2.00	2.00
chr20-8901543-8903090	37.00	37.00
chr20-33361770-33363718	16.00	16.00

Continued on next page

Sample	BLAT	PxBLAT
chr20-60319529-60322435	17.00	17.00
chr20-9417083-9419320	19.00	19.00
chr20-47368389-47371245	181.00	181.00
chr20-49829087-49831301	97.00	97.00
chr20-45577205-45579659	30.00	30.00
chr20-9885847-9887724	4.00	4.00
chr20-34086336-34087450	25.00	25.00
chr20-12690873-12693547	25.00	25.00
chr20-61265483-61267506	31.00	31.00
chr20-60767473-60770234	21.00	21.00
chr20-37096290-37097735	46.00	46.00
chr20-19806699-19808018	39.00	39.00
chr20-5383554-5385658	41.00	41.00
chr20-46475786-46476831	62.00	62.00
chr20-35346166-35347602	30.00	30.00
chr20-39431060-39432816	28.00	28.00
chr20-50643697-50644883	205.00	205.00
chr20-22403148-22404794	15.00	15.00
chr20-26331430-26334030	3.00	3.00
chr20-14375436-14376511	196.00	196.00
chr20-32839818-32840903	124.00	124.00
chr20-20727967-20730763	59.00	59.00
chr20-25447522-25449068	21.00	21.00
chr20-52094840-52096680	143.00	143.00
chr20-21563954-21565882	146.00	146.00
chr20-5436550-5437659	70.00	70.00
chr20-63482588-63484649	191.00	191.00
chr20-19772825-19774949	43.00	43.00
chr20-38393781-38395769	19.00	19.00
chr20-34751103-34754059	175.00	175.00
chr20-26987677-26990651	24.00	24.00
chr20-8530344-8531955	41.00	41.00
chr20-2975561-2977607	0.00	0.00
chr20-56181870-56183051	0.00	0.00
chr20-35814820-35817469	0.00	0.00
chr20-13176813-13178454	0.00	0.00
chr20-45598592-45599763	0.00	0.00
chr20-23037040-23038387	0.00	0.00
chr20-17554036-17556666	0.00	0.00
chr20-59732533-59734709	0.00	0.00
chr20-43253525-43256343	0.00	0.00
chr20-5541509-5543311	0.00	0.00

Continued on next page

Sample	BLAT	PxBLAT
chr20-48761025-48762893	0.00	0.00
chr20-34464744-34466642	0.00	0.00
chr20-42866256-42868185	0.00	0.00
chr20-50226595-50228543	0.00	0.00
chr20-43868259-43870002	0.00	0.00
chr20-61460131-61461808	0.00	0.00
chr20-18061681-18062812	0.00	0.00
chr20-33365416-33368097	0.00	0.00
chr20-15504260-15505833	0.00	0.00
chr20-14468799-14471029	0.00	0.00
chr20-3363803-3366253	0.00	0.00
chr20-15709653-15710930	0.00	0.00
